# Effects of Dexmedetomidine on motor- and somatosensory-evoked potentials in patients with thoracic spinal cord tumor: a randomized controlled trial

**DOI:** 10.1186/s12871-016-0217-y

**Published:** 2016-08-02

**Authors:** Yan Li, Lingzhong Meng, Yuming Peng, Hui Qiao, Lanjun Guo, Ruquan Han, Adrian W. Gelb

**Affiliations:** 1 Department of Anesthesiology, Beijing Tiantan Hospital, Capital Medical University, No. 6 Tiantan Xili, Dongcheng District Beijing, China 100050; 2 Neurophysiological Monitoring, Beijing Tiantan Hospital, Capital Medical University, Beijing, China; 3 Departments of Anesthesia and Perioperative Care, University of California San Francisco, San Francisco, CA USA; 4 Neurosurgery/Neurophysiological Monitoring Service, University of California San Francisco, San Francisco, CA USA

**Keywords:** Motor-evoked potentials, Somatosensory-evoked potentials, Dexmedetomidine, RCT, Spinal cord tumor

## Abstract

**Background:**

We hypothesized that the addition of dexmedetomidine in a clinically relevant dose to propofol-remifentanil anesthesia regimen does not exert an adverse effect on motor-evoked potentials (MEP) and somatosensory-evoked potentials (SSEP) in adult patients undergoing thoracic spinal cord tumor resection.

**Methods:**

Seventy-one adult patients were randomized into three groups. Propofol group (*n* = 25): propofol-remifentanil regimenand the dosage was adjusted to maintain the bispectral index (BIS) between 40 and 50. DP adjusted group (*n* = 23): Dexmedetomidine (0.5 μg/kg loading dose infused over 10 min followed by a constant infusion of 0.5 μg/kg/h) was added to the propofol-remifentanil regimen and propofol was adjusted to maintain BIS between 40 and 50. DP unadjusted group (*n* = 23): Dexmedetomidine (administer as DP adjusted group) was added to the propofol-remifentanil regimen and propofol was not adjusted. All patients received MEP, SSEP and BIS monitoring.

**Results:**

There were no significant changes in the amplitude and latency of MEP and SSEP among different groups (*P* > 0.05). The estimated propofol plasma concentration in DP adjusted group (2.7 ± 0.3 μg/ml) was significantly lower than in propofol group (3.1 ± 0.2 μg/ml) and DP unadjusted group (3.1 ± 0.2 μg/ml) (*P* = 0.000). BIS in DP unadjusted group (35 ± 5) was significantly lower than in propofol group (44 ± 3) (*P* = 0.000).

**Conclusions:**

The addition of dexmedetomidine to propofol-remifentanil regimen does not exert an adverse effect on MEP and SSEP monitoring in adult patients undergoing thoracic spinal cord tumor resection.

**Trial registration:**

The study was registered with the Chinese Clinical Trial Registry on January 31st, 2014. The reference number was ChiCTR-TRC-14004229.

## Background

Motor-evoked potential (MEP) and somatosensory-evoked potential (SSEP) monitoring are widely used during spinal surgeries, which threat the integrity of motor and sensory pathways in the spinal cord. The effect of anesthetic agents on the amplitude and latency of MEP and SSEP monitoring is dose-dependent [1–3]. Total intravenous anesthesia (TIVA) with propofol and opioid is commonly recommended for surgeries that require MEP and SSEP monitoring [1, 4]. However, even propofol used with a large dose can also affect MEP monitoring [5].

Dexmedetomidine is a potent and highly selective alpha-2 agonist. It has the effect of sedation, analgesia, sympatholysis, minimal respiratory depression and possible neuroprotection [6–9]. Its addition to the anesthetic regimen is believed to have the potential of sparing other hypnotics requirement, especially propofol, thus facilitating MEP and SSEP monitoring while providing the beneficial effects it has. Previous studies examining the effect of dexmedetomidine on MEP and SSEP monitoring claimed both a null [4, 10–14] and significant adverse effect [11, 15]. The design of most studies was longitudinal in which patients served as their own controls [10–13]. Therefore, the results may be confounded by the carryover or top-up effect after the addition of dexmedetomidine. The only randomized controlled trial used etomidate instead of propofol for anesthetic maintenance and this is not a currently recommended practice [4].

Another consideration is that all the previous studies were done in patients suffering from various non-tumor spinal conditions. In patients diagnosed with spinal cord tumor, the combination of dexmedetomidine may affect MEP and SSEP monitoring differently compared with surgery on the bony structures only because the neural pathways may have been adversely injured by the tumor [16, 17].

In this study we propose our hypothesis that the combination of dexmedetomidine in a clinically relevant dose to an anesthetic regimen reduces propofol and remifentanil requirement does not exert an adverse effect on MEP and SSEP monitoring in patients with thoracic spinal cord tumors.

## Methods

This single-center and randomized study was conducted in Beijing Tiantan Hospital, Capital Medical University. The study was approved by the Ethics Committee of Beijing Tiantan Hospital, Capital Medical University (reference number was ky-2010-018-02). Written informed consent was obtained from all participants. The study was registered with the Chinese Clinical Trial Registry (the reference number was ChiCTR-TRC-14004229).

### Patients

We recruited patients scheduled for elective thoracic spinal cord tumor resection based on magnetic resonance imaging (MRI) studies. Other inclusion criteria included age between 18 and 60 years old and American Society of Anesthesiologists (ASA) physical status I or II. The patients who were pregnant and/or lactating, chronic using or addiction of analgesics were excluded from the study. The patients who were with illegal drug or alcohol abuse, obesity (BMI ≥ 30 kg/m^2^), anemia (hemoglobin < 11 g/dl), and major organ dysfunctions were also excluded from the study.

### Grouping

Patients who met the recruitment criteria were randomly assigned to one of the three study groups, labeled “propofol group”, “DP adjusted group”, or “DP unadjusted group”. Randomization was based on a computer generated random digits table (SPSS Inc., Chicago, IL). Permuted-block randomization was used with a block size of 3 and an allocation ratio of 1:1:1. Since anesthesiologist was in charge of the anesthesia management and all evaluation of outcomes were based on the result of electrophysiological monitoring, the anesthesiologist could not be blinded. Neurophysiologists and patients were blinded to the study group till the evaluation finished. In propofol group, the anesthesia was maintained using propofol and remifentanil infusions only. The blood plasma concentration of remifentanil was fixed at 4 ng/ml while the propofol infusion was adjusted to maintain the BIS measurement between 40 and 50. In DP adjusted group, dexmedetomidine (0.5 μg/kg loading dose infused over 10 min followed by a constant infusion rate of 0.5 μg/kg/h) was added to propofol and remifentanil infusions. While the dexmedetomidine infusion was standardized and the blood plasma concentration of remifentanil was fixed at 4 ng/ml while the propofol infusion was adjusted to maintain the BIS measurement between 40 and 50 for 90 % of anesthesia time and no deviations for more than 5 min. In DP unadjusted group, the anesthesia was maintained using propofol and remifentanil infusions as in propofol group. After BIS was maintained between 40 and 50, dexmedetomidine was then added (0.5 μg/kg loading dose infused over 10 min followed by a constant infusion rate of 0.5 μg/kg/h). The propofol was not adjusted as in DP unadjusted group.

### Anesthesia and management

The patients did not receive premedication. Anesthesia was induced by propofol (5 μg/ml) infusion managed with a Diprifusor propofol infusion device (Marsh model, Master Target Control infusion system, Fresenius-Vial, Brezins, France), remifentanil (4 ng/ml) infusion managed with an infusion device (Minto model CP-600TCI, Beijing Slgo Medical Technology Co., Ltd., Beijing, China) and rocuronium (0.6 mg/kg). Then patients received endotracheal intubation and mechanical ventilation adjusting to maintain the end-tidal carbon dioxide between 35 and 40 mmHg. In addition to the routine ASA monitors, patients received MEP (Cascade, Cadwell Laboratories Inc, WA, USA), SSEP (Cascade, Cadwell Laboratories Inc, WA, USA), bispectral index (BIS) (BIS™, Covidien, San Jose, CA, USA) and intra-arterial blood pressure monitoring. Bradycardia, defined as the heart rate (HR) < 50 bpm, was treated with atropine (0.5 mg) bolus administration. Hypotension, defined as a decreasing of mean arterial pressure (MAP) more than twenty percentage of the baseline, was treated by using a dopamine infusion titrated. The baseline blood pressure value was determined based on preoperative evaluation on the day before surgery.

### Motor- and somatosensory-evoked potential monitoring

MEP was recorded from paired needle electrodes placed bilaterally in the tibias anterior and extensor digital muscles, and hand muscles. Electrical current delivered to corkscrew electrodes were inserted at the C3 and C4 sites (international 10–20 system) with train of six to nine pulses, 300 to 500 volts, 75 ms interval pause (ISI) and one to four microsecond of inter-stimulus interval using Cadwell Cascade neurophysiologic monitoring system (Cascade, Cadwell Laboratories Inc, WA, USA). Anodal stimulation was applied to trigger contralateral MEP responses. The number of pulses and voltage were established at baseline and maintained throughout the surgery. Surface-stimulating electrodes for SSEP monitoring were placed over each ulnar nerve at the wrists and over each posterior tibial nerve at the ankles. Needle electrodes were placed over the somatosensory hand cortex at scalp sites C3′-Fz, C4′-Fz, Cz′-Fz and C3′-C4′ to record the primary cortical responses. Ulnar and posterior tibial nerves were stimulated synchronously at 2.79/s and averaged over 500 stimuli. Ulnar nerves were stimulated at 15 mA, and posterior tibial nerves were stimulated at 25 mA. Then europhysiologists were blinded to the group till the evaluation finished.

**Fig. 1:**
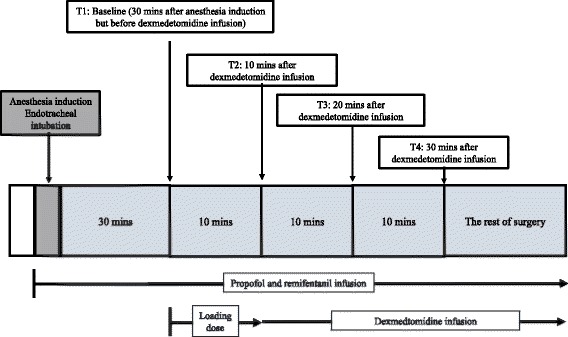
Flow chart of study protocol

Prior to surgery, the muscle strength of the left and right lower extremity was assessed by the attending neurosurgeon who was blinded to the randomization using a 0-2-5 scale, with 5 indicating normal strength and 0 complete paralysis. MEP, SSEP, BIS, mean arterial pressure (MAP) and heart rate (HR) were measured at 4 different time points (Fig. 1). T1 was the baseline value obtained 30 min after anesthesia induction and endotracheal intubation but before the dexmedetomidine infusion. T2 was 10 min after T1 when the dexmedetomidine loading dose infusion (0.5 μg/kg over 10 min) was just finished in DP adjusted group and DP unadjusted group. All variables were recorded again when the dexmedetomidine maintenance infusion (0.5 μg/kg/h) has lasted for 10 (T3) and 20 (T4) minutes, respectively, in both groups. The muscle relaxation related to rocuronium was reversed using neostigmine (0.05–0.07 mg/kg) if necessary before the first measurement. A T4/T1 90 % recovery of the muscle strength based on the train-of-four ratio was deemed acceptable for the study. All measurements were done with the patient in the lateral position and prior to skin incision in order to avoid the confounding effect of surgical manipulation on MEP and SSEP monitoring. More than 50 % decrease of the amplitude and more than 10 % prolongation of the latency of both SSEP and MEP monitoring from the baseline values were defined as clinically meaningful changes [18].

### Statistical analysis

Sample size calculation was performed by PASS 2008 software (NCSS LLC, USA) for windows. Based on the study in which used MEP amplitude (500 μV; SD,110) to detect a 50 % difference in MEP amplitude (decrease from 500 to 250 μV) [13], 20 patients were required to detect an effect size of 0.8, assuming a power of 80 % and a 2-sided α level of 5 %. Considering the possibility of early termination during the study and 20–30 % for a drop-out rate, therefore 30 patients were recruited in each group.

Statistical analyses were performed using the Statistical Package for Social Sciences (Version 19.0, SPSS Inc., Chicago, IL). All quantitative data were analyzed for normal distribution and homogeneity of variance. Data that showed a normal distribution were presented as the means ± SD. The non-normal distribution data were presented as median. One-way analysis of variance (ANOVA) of repeated measurements was used to evaluate differences between the means at different time points in one group. Variables between groups underwent Student-Newman-Keuls tests with multiple comparison correction. All *P* values were two sided, and α level of 0.05 was considered statistically significant.

## Results

**Fig. 2:**
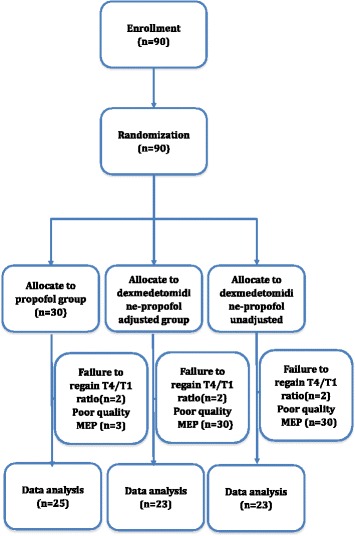
Flow chart of patient selection

Ninety patients were equally enrolled in this study. 19 patients were excluded from analysis due to either poor quality MEP monitoring (3, 5, 5 in 3 groups respectively) or failure to regain T4 ratio which defined as T4/T1 recovered less than 90 % based on the train-of-four ratio after the intubation (2 in each group). The patients’ recruitment was showed in Fig. 2. There were no significant differences of age, gender, height, and weight among groups (Table 1). There were no significant differences in the muscle strength of the left and right lower extremities among different groups (Table 2).

**Table 1:** Demographic data (mean ± SD)

Group	Sex (male/female)	Age (year)	Height (cm)	Weight (kg)
Propofol group (*n* = 25)	18/7	40 ± 11	169 ± 7	68 ± 11
DP adjusted group (*n* = 23)	12/11	40 ± 14	164 ± 7	65 ± 10
DP unadjusted group (*n* = 23)	13/10	41 ± 13	166 ± 9	63 ± 13

**Table 2:** Muscle strength of the lower extremities before surgery (mean ± SD)

Group	Left	P	Right	P
Propofol group (*n* = 25)	4.3 ± 1.0		4.2 ± 1.0	
DP adjusted group (*n* = 23)	4.1 ± 1.1	0.72	4.1 ± 1.1	0.82
DP unadjusted group (*n* = 23)	4.2 ± 1.1	0.83	4.3 ± 1.1	0.74

P: Comparison with propofol group

The estimated blood plasma concentration of propofol in DP adjusted group (2.7 ± 0.3 μg/ml) was significantly lower than in propofol group and DP unadjusted group (3.1 ± 0.2 μg/ml and 3.1 ± 0.2 μg/ml) (*P* = 0.000) (Table 3).

**Table 3:** Estimated plasma concentration of propofol (μg/ml, mean ± SD) at different time points

Group	T1(μg/ml)	P	T2(μg/ml)	P	T3(μg/ml)	P	T4(μg/ml)	P
Propofol group (*n* = 25)	3.1 ± 0.2		3.1 ± 0.2		3.1 ± 0.2		3.1 ± 0.2	
DP adjusted group (*n* = 23)	3.0 ± 0.2	0.313	2.8 ± 0.2^a^	0.003	2.7 ± 0.3*^a^	0.000	2.7 ± 0.3*^a^	0.000
DP unadjusted group (*n* = 23)	3.1 ± 0.2	0.829	3.1 ± 0.2	0.829	3.1 ± 0.2	0.939	3.1 ± 0.2	0.939

* *P* = 0.01 (comparison among different time points)

^a^Comparison with propofol group

There were no significant differences in MAP and HR among different measurement points in propofol group (Table 4). In DP adjusted group (93 ± 14 mmHg) and DP unadjusted group (94 ± 10 mmHg) the MAP was significantly higher than propofol group (86 ± 15 mmHg) (*P* = 0.037, 0.032). In DP unadjusted group, the HR at T2-T4 (59 ± 6, 59 ± 5, 60 ± 6pbm) was lower than T1 (66 ± 8pbm) (*P* = 0.000, 0.000, 0.003, respectively).

**Table 4:** MAP, HR, BIS at different time points (mean ± SD)

Variable	Group	T1	P	T2	P	T3	P	T4	P
MAP (mmHg)	Propofol group (*n* = 25)	86 ± 11		86 ± 15		86 ± 13		84 ± 12	
	DP adjusted group (*n* = 23)	80 ± 11	0.061	93 ± 14**^a^	0.037	95 ± 15**^a^	0.019	95 ± 12**^a^	0.003
	DP unadjusted group (*n* = 23)	80 ± 10	0.098	94 ± 10**^a^	0.032	95 ± 13**^a^	0.019	95 ± 11**^a^	0.003
HR (bpm)	Propofol group (*n* = 25)	65 ± 10		60 ± 7		59 ± 6		60 ± 8	
	DP adjusted group (*n* = 23)	64 ± 10	0.541	59 ± 9	0.464	58 ± 9*	0.816	59 ± 8	0.626
	DP unadjusted group (*n* = 23)	66 ± 8	0.841	59 ± 6**	0.489	59 ± 5**	0.842	60 ± 6*	0.850
BIS	Propofol group (*n* = 25)	45 ± 3		44 ± 3		44 ± 3		42 ± 8	
	DP adjusted group (*n* = 23)	44 ± 4	0.393	43 ± 3	0.146	43 ± 3	0.428	43 ± 3	0.534
	DP unadjusted group (*n* = 23)	45 ± 3	0.732	37 ± 6**^a^	0.000	35 ± 5**^a^	0.000	35 ± 6**^a^	0.000

*P < 0.05, **P = 0.000 (comparison among different time points)

^a^Comparison with propofol group

There were no significant differences in the BIS of propofol groups and DP adjusted group (Table 4). However, the BIS measured at points T2-T4 in DP unadjusted group (37 ± 6, 35 ± 5, 35 ± 6) was lowered than the corresponding points in propofol group (44 ± 3, 44 ± 3, 42 ± 8) (*P* = 0.000).

There were no significant changes in the amplitude and latency of both MEP (Table 5) and SSEP (Table 6) among different time pointsin all 3 groups (*P* > 0.05). There were also no significant differences of the measurements among different groups (*P* > 0.05).

**Table 5:** Motor-evoked potential at different time points (mean ± SD)

Group	Measurement	T1	T2	T3	T4
Propofol group (*n* = 25)	ULA (uv)	280 ± 148	307 ± 147	326 ± 154	326 ± 169
	ULL (ms)	20.7 ± 3.2	21.3 ± 2.2	21.6 ± 2.4	21.9 ± 2.3
	URA (uv)	323 ± 139	307 ± 138	366 ± 164	343 ± 145
	URL (ms)	21.2 ± 3.6	22.2 ± 4.0	21.8 ± 2.7	21.6 ± 3.5
	LLA (uv)	152 ± 84	177 ± 82	208 ± 141	221 ± 113
	LLL (ms)	38.9 ± 4.8	40.1 ± 5.7	40.2 ± 6.9	39.4 ± 6.6
	LRA (uv)	127 ± 91	121 ± 84	140 ± 90	131 ± 105
	LRL (ms)	39.0 ± 9.9	39.4 ± 10.6	40.4 ± 11.1	39.6 ± 4.2
DP adjusted group (*n* = 23)	ULA (uv)	293 ± 131	341 ± 207	313 ± 155	313 ± 178
	ULL (ms)	20.8 ± 2.1	21.8 ± 3.6	21.3 ± 3.6	20.9 ± 2.8
	URA (uv)	256 ± 137	302 ± 193	303 ± 158	339 ± 206
	URL (ms)	21.1 ± 2.6	20.6 ± 2.8	20.9 ± 3.0	21.5 ± 3.3
	LLA (uv)	174 ± 135	185 ± 129	179 ± 106	153 ± 78
	LLL (ms)	39.4 ± 5.2	39.5 ± 3.6	39.2 ± 3.2	37.3 ± 9.9
	LRA (uv)	154 ± 77	173 ± 153	187 ± 137	160 ± 98
	LRL (ms)	40.5 ± 3.9	40.6 ± 4.1	40.8 ± 5.0	41.3 ± 5.3
DP unadjusted group (*n* = 23)	ULA (uv)	354 ± 179	353 ± 116	343 ± 153	355 ± 158
	ULL (ms)	21.4 ± 2.3	21.3 ± 2.4	22.1 ± 4.0	23.1 ± 3.8
	URA (uv)	354 ± 161	351 ± 191	316 ± 197	292 ± 163
	URL (ms)	21.6 ± 2.6	21.7 ± 3.0	22.4 ± 4.0	23.1 ± 4.1
	LLA (uv)	171 ± 114	196 ± 171	215 ± 178	169 ± 144
	LLL (ms)	41.2 ± 4.4	40.0 ± 4.2	39.8 ± 3.5	39.9 ± 3.6
	LRA (uv)	206 ± 153	207 ± 192	225 ± 177	199 ± 138
	LRL (ms)	38.4 ± 10.7	39.8 ± 4.2	40.7 ± 4.7	38.2 ± 10.0

*ULA* amplitude of left upper limb, *ULL* latency of left upper limb, *URA* amplitude of right upper limb, *URL* latency of right upper limb, *LLA* amplitude of left lower limb, *LLL* latency of left lower limb, *LRA* amplitude of right lower limb, *LRL* latency of right lower limb

P: Comparison with propofol group

**Table 6:** Somatosensory-evoked potential at different time points (mean ± SD)

Group	Measurement	T1	T2	T3	T4
Propofol group (*n* = 25)	ULA (uv)	2.0 ± 0.9	1.9 ± 0.7	1.8 ± 0.9	2.1 ± 1.0
	ULL (ms)	20.1 ± 0.8	20.3 ± 1.2	20.5 ± 1.4	20.6 ± 1.5
	URA (uv)	2.4 ± 1.4	2.1 ± 1.1	2.3 ± 1.3	1.9 ± 1.1
	URL (ms)	20.2 ± 1.4	20.2 ± 1.3	20.5 ± 1.3	20.6 ± 1.6
	LLA(uv)	1.1 ± 1.1	1.0 ± 0.9	1.1 ± 0.8	1.1 ± 0.9
	LLL (ms)	39.6 ± 4.0	40.1 ± 4.2	40.2 ± 4.3	39.8 ± 4.6
	LRA(uv)	1.3 ± 1.2	1.6 ± 2.2	1.0 ± 1.0	0.8 ± 0.9
	LRL (ms)	40.8 ± 4.6	40.7 ± 4.5	40.9 ± 4.5	40.7 ± 4.7
DP adjusted group (*n* = 23)	ULA (uv)	2.0 ± 1.1	2.1 ± 1.1	2.0 ± 1.1	2.0 ± 1.1
	ULL (ms)	19.2 ± 1.5	19.4 ± 1.6	19.7 ± 1.6	19.7 ± 1.7
	URA (uv)	2.1 ± 1.4	2.1 ± 1.3	2.3 ± 1.2	2.3 ± 1.3
	URL (ms)	19.8 ± 1.8	19.8 ± 1.5	20.0 ± 1.5	19.9 ± 1.5
	LLA(uv)	1.5 ± 1.5	1.5 ± 1.6	1.8 ± 2.0	1.5 ± 1.2
	LLL(ms)	39.7 ± 4.4	40.4 ± 4.1	40.9 ± 4.8	41.2 ± 4.1
	LRA(uv)	1.4 ± 1.6	1.6 ± 1.8	1.6 ± 2.0	1.5 ± 1.8
	LRL (ms)	38.8 ± 3.8	40.0 ± 3.3	40.1 ± 3.2	39.6 ± 2.4
DP unadjusted group (*n* = 23)	ULA (uv)	1.6 ± 1.0	1.7 ± 1.0	1.7 ± 1.1	1.8 ± 1.1
	ULL (ms)	19.2 ± 2.1	19.4 ± 2.2	19.6 ± 2.4	19.2 ± 3.1
	URA(uv)	1.6 ± 1.0	1.7 ± 0.9	1.5 ± 0.9	1.6 ± 0.8
	URL(ms)	19.8 ± 2.7	20.1 ± 2.6	20.3 ± 2.3	20.1 ± 2.7
	LLA(uv)	1.1 ± 1.0	0.9 ± 0.7	1.2 ± 1.0	1.3 ± 1.0
	LLL(ms)	39.8 ± 4.3	39.9 ± 3.9	40.6 ± 4.1	41.0 ± 4.3
	LRA(uv)	1.0 ± 0.7	0.7 ± 0.4	1.1 ± 0.7	1.3 ± 1.0
	LRL (ms)	39.8 ± 4.1	40.2 ± 4.1	41.3 ± 4.5	41.1 ± 4.3

*ULA* amplitude of left upper limb, *ULL* latency of left upper limb, *URA* amplitude of right upper limb, *URL* latency of right upper limb, *LLA* amplitude of left lower limb, *LLL* latency of left lower limb, *LRA* amplitude of right lower limb, *LRL* latency of right lower limb

P: comparison with propofol group

## Discussion

This randomized controlled study in patients with thoracic spinal cord tumors shows that the addition of dexmedetomidine to a TIVA regimen comprising of propofol and remifentanil does not adversely affect the MEP and SSEP monitoring, and the addition of dexmedetomidine reduces the propofol requirements for a comparable BIS measurement. Moreover, the deepening of anesthesia via propofol infusion does not necessarily affect the MEP and SSEP monitoring.

All major hypnotic agents used in anesthesia can exert dose-dependent adverse effect on MEP and SSEP monitoring [1–3]. Inhalational anesthetic agents have a potent effect and therefore are either avoided or used in low concentrations during MEP monitoring [1–4]. Propofol at higher concentrations can also depress the amplitude of the MEP [5]. Currently, the anesthetic technique that is frequently recommended during MEP and SSEP monitoring is a TIVA regimen comprising of multiple drugs at lower doses than usually used. The rationale of this strategy is to provide an adequate and balanced anesthesia via a combination of drugs that have different but supplementary mechanisms while minimizing the adverse effect on evoked potential monitoring of an otherwise large dose if the drug is used alone. This strategy is typically executed via a propofol-opioid, either remifentanil or fentanyl, combination [1, 4].

The multiple advantageous properties of dexmedetomidine make it a potentially useful drug for inclusion in a TIVA regimen in monitored cases [11–14]. The rationale for adding dexmedetomidine is to reduce the dose of propofol or to deepen the anesthesia without increasing the propofol infusion rate, in addition to potentiation of the opioid analgesia [11–14]. Previous studies suggested that the effect of dexmedetomidine on MEP and SSEP monitoring is dose-dependent with the drug blood concentration of 0.3–0.6 ng/ml producing no adverse effect [10] while that of 0.6–0.8 ng/ml causing a significant attenuation of the MEP amplitude [11]. However, these results are difficult to consolidate because one study used propofol-remifentanil [12] while the other desflurane-remifentanil [10]. Moreover, the designs of these studies were heterogeneous including drug titration and patient populations. The major difference between our study and previously study is that we used arandomized controlled study. Our results show that the addition of dexmedetomidine to a TIVA regimen does not adversely affect the MEP and SSEP monitoring. In contrast, Mahmoud et al. reported two cases of loss of MEP amplitude during pediatric spine surgery with dexmedetomidine [15]. One case was an obese child, propofol and dexmedetomidine were calculated on actual rather than lean body mass. It is therefore possible that the patient had higher serum concentration of both drugs. In the second patient, decreased MEP amplitude was monitored after a bolus of 1 μg/kg dexmedetomidine was administered over 10 min. It is possible that the combination of dexmedetomidine and propofol might have a cumulative suppressing effect on MEP. Our study included patients between 18 and 60 years old and the dose of dexmedetomidine we use that would approximate the plasma levels achieved by Bala et al. [10]. Even in DP unadjusted group, the deepening of anesthesia via propofol infusion does not necessarily affect the MEP and SSEP monitoring.

This study has limitations. First, a fixed dose of dexmedetomidine, which is 0.5 μg/kg loading dose for 10 min followed by a 0.5 μg/kg/h infusion, didn’t ensure a constant level of its plasma concentration. Second, we recruited patients scheduled for elective thoracic spinal cord tumor resection. Poor signal of MEP and SSEP is inavoidable in such patients. Malhotra [16] reported that ability to achieve intraoperative monitor baseline data varied from 70 to 98 % for SSEP and 66 to 100 % for MEP in absence of neural axis abnormality. In this study, the total excluded number in three groups are respectively 5/30, 7/30, 7/30 respectively. Although the high percent of failure rate may lead to a selection bias, the remaining patients in each group are still comparable to study the effects of dexmedetomidine in a clinically relevant dose on MEP and SSEP.

## Conclusion

The addition of dexmedetomidine in a commonly used clinical dose to a propofol-remifentanil regimen does not exert an adverse effect on MEP and SSEP monitoring in adult patients undergoing thoracic spinal cord tumor resection. In this patient population, dexmedetomidine could be added to the TIVA regimen without altering the propofol dose thereby deepening the anesthesia without adversely influencing MEP and SSEP monitoring.

## Abbreviations

MEP: motor-evoked potentials; SSEP: somatosensory-evoked potentials; BIS: bispectral index; TIVA: total intravenous anesthesia; MRI: magnetic resonance imaging; ASA: American Society of Anesthesiologists; MAP: mean arterial pressure; HR: heart rate
